# The *Brucella* Effector Protein BspF Crotonylates TRIM38 to Inhibit NF-κB and MAPK Signaling Pathway

**DOI:** 10.3390/ijms26083573

**Published:** 2025-04-10

**Authors:** Huan Zhang, Yukai Xing, Jinying Zhu, Sijiao Wu, Jingbo Gao, Yuqi Wang, Ze Yu, Ang Li, Yuzhuo Li, Xiaoyue Chen, Zeliang Chen

**Affiliations:** 1 Key Laboratory of Livestock Infectious Disease, Ministry of Education, Shenyang Agricultural University, Shenyang 110866, China; zhanghuan1988@syau.edu.cn (H.Z.); xingyu18404243003@outlook.com (Y.X.); jean_zz512@163.com (J.Z.); 15035103947@163.com (S.W.); 13614174575@163.com (J.G.); wangww0093@163.com (Y.W.); m15140968823@163.com (Z.Y.); liang000624@outlook.com (A.L.); 19802486746@163.com (Y.L.); 2 Department of Epidemiology, School of Public Health, Sun Yat-sen University, Guangzhou 510275, China

**Keywords:** *Brucella*, BspF, crotonylation, NF-κB, MAPK

## Abstract

The type IV secretion system (T4SS) is an important virulence factor of *Brucella*. T4SS secretes 16 effector proteins, which affect the intracellular transport of *Brucella*-containing vacuoles and regulate the host immune response, helping *Brucella* survive and replicate in host cells. In our previous crotonylation proteomics data of HEK-293T cell proteins triggered by BspF, we found BspF crotonylated on TRIM38, which is an important modulator in the pathways of inflammation, and the crotonylation site is K142. Therefore, it is speculated that BspF may be involved in the regulation of host inflammatory response during *Brucella* infection. In this study, we found that BspF-mediated TRIM38K142 crotonylation promotes the ubiquitination of tumor necrosis factor receptor (TNFR)-associated factor 6 (TRAF6), leading to the degradation of TRAF6 and thereby inhibiting the transduction of Nuclear factor-kappaB (NF-κB), p38 Mitogen-activated protein kinase (MAPK), and c-Jun N-terminal kinases (JNK) MAPK signaling pathways and the secretion of pro-inflammatory factors *IL-6* and *IL-8*, which finally helps *Brucella* promote intracellular survival. This study provides a new theoretical basis for the intracellular survival of host innate immunity through the T4SS, provides new insights into the pathogenic mechanism and treatment of *Brucella*, and provides an important reference for the study of non-histone crotonylation function.

## 1. Introduction

*Brucella* is a Gram-negative intra-encapsulated parasitic bacterium in the form of short rods or spheres, which invades the body mainly through the skin or the mucous membrane of the digestive tract. Brucellosis leads to miscarriage, stillbirth, and sterility through attacking the reproductive system of animals and can also reduce livestock production and service capacity, resulting in significant economic losses [1,2,3]. Among different types of *Brucella*, the most common and dangerous strains are *B. melitensis*, *B. abortus*, and *B. suis* [4]. To reduce the impact of brucellosis, a gene-deletion marker vaccine strain with favorable safety and immunogenicity has been developed. It has a lower risk of virulence reversion compared to attenuated live vaccines, offering novel strategies for the prevention and control of brucellosis [5].

Although *Brucella* lacks classical virulence factors such as exotoxins, secreted proteases, cytolysins, and virulence plasmids, it contains lipopolysaccharides (LPSs), the type IV secretion system (T4SS), cyclic β-1-2-glucan (CβG), and other virulence factors. After *Brucella* infection, the human body usually has a latent period of 2–4 weeks before symptoms appear, which is attributed to the ability of *Brucella* to evade the clearance of innate immunity in a variety of ways on the mucosal surface and during systemic infection [6]. The T4SS is an important *Brucella* virulence factor, which secretes effector proteins into the host and interferes with key signaling pathways of host cells, thereby shielding *Brucella* from phagocytosis and elimination by host cells and intracellular lysosomes, affecting the maturation of *Brucella* vesicles, interfering with normal host immunity, and assisting *Brucella* to complete intracellular invasion, colonization, and massive proliferation [7]. T4SS effectors contain Bsp (*Brucella*-secreted protein) A, B, C, E, F, and L, Btp (*Brucella* TIR protein) A and B, VirB-coregulated effector (Vce) A and C, BPE (*Brucella* protein effector, BPE) 005, 123, 043, and 275, Rab2 interacting conserved protein A (RicA), and secreted effector protein A (SepA). BspF contains a Gcn5-related N-acetyltransferase (GNAT) family acetyltransferase domain, which regulates host secretory function together with BspA and BspB to promote intracellular bacterial growth [8]. BspL enhances Endoplasmic Reticulum-Associated Degradation (ERAD) at the late stages of infection, and BspL targeting of Herp and ERAD allows tight control of the kinetics of autophagic *Brucella*-containing vacuole formation, delaying the last step of its intracellular cycle and cell-to-cell spread [9]. In addition, new studies have shown that BspF can target endosome recycling to inhibit the reverse transport of the trans-Golgi network (TGN), disrupting Arf6/Rab8a-dependent transport in circulating endosomes, and specifically promote *Brucella* replication in rBCV [10]. Our previous study also found that BspF has de-histone crotonyltransferase activity and can regulate the global crotonylation level in cells [11].

Lysine crotonylation is enzymatically regulated by the dynamic balance between crotonyltransferases and de-crotonyltransferases [12]. Both histone and non-histone can undergo crotonylation, and histones are crotonylated and participate in many biological processes by regulating chromatin remodeling, metabolism, cell cycle, and cell organization. Current studies have shown that crotonylation on histones can promote gene transcription and spermatogenesis [13,14]. However, the function and mechanism of non-histone crotonylation is still unclear. In addition, crotonylation also plays a role in diseases. It is reported that in liver, stomach, and kidney cancers, the levels of crotonylation were down-regulated, while being up-regulated in thyroid, esophageal, colon, pancreatic, and lung cancers, suggesting that crotonylation may regulate different cancer progression [15,16,17,18]. Furthermore, crotonyltransferase promotes human immunodeficiency virus (HIV) histone crotonylation, which in turn regulates HIV transcription, leading to latent HIV viral reactivation and participates in the establishment of HIV latency, and histone crotonylation may serve as a potential therapeutic target for HIV eradication [19,20,21].

As an intracellular parasite, *Brucella* deploys a plethora of virulence factors that enable it to persist within the host environment [22]. The type IV secretion system is an important virulence factor that has been extensively researched and identified as a crucial mechanism facilitating *Brucella*’s long-term survival. Key functions of the T4SS encompass regulating the intracellular trafficking of *Brucella*-containing vacuoles in host macrophages and allowing these bacteria to evade phagocytic degradation in lysosomes [23]. Another way that the T4SS promotes virulence is by regulating the host immune responses [24]. As an effector protein secreted by the T4SS, BspF is indispensable for the replication of *Brucella* within host cells. While BspF facilitates the intracellular proliferation of *Brucella* in replicative *Brucella*-containing vacuoles (rBCVs), it does not enhance the biosynthesis of rBCVs [10]. BspF selectively targets endosome recycling, impeding retrograde transport to the trans-Golgi network (TGN), and interacts with the Arf6 GTPase activator protein (GAP) ACAP1, thereby enhancing bacterial growth [10].

Previous studies have found that the *Brucella* effector BspI can interact with IRE1 in HeLa cells, inhibit the activity of IRE 1 kinase to inhibit the induction of pro-inflammatory cytokines, and thus control the occurrence of inflammation [25]. So, we speculated whether BspF also had a similar function. In our previous proteomic investigation of crotonylation, we identified BspF-mediated crotonylation on TRIM38K142 [11]. Herein, we further validate this crotonylation of TRIM38 by BspF. TRIM38 possesses well-conserved RING (ring finger domain) (16–63 aa), B-BOX (88–129 aa), and PRY/SPRY (274–465 aa) domains that execute diverse functions. TRIM38 has been previously demonstrated in RAW264.7 cells to negatively modulate Toll-like receptor (TLR) 4/6-driven NF-κB activation by targeting TRAF3 for proteasomal degradation [26]. In addition, TRIM38 can interact with TIR domain-containing adaptor inducing 1FN-β (TRIF), triggering its K48 polyubiquitination via E3 ubiquitin ligase activity, leading to TRIF degradation [27]. Moreover, TRIM38′s PRY/SPRY domain has been shown to interact with TRAF6, facilitate K48 polyubiquitination of TRAF6, prompt its proteasomal degradation, and thereby attenuate TLR-driven NF-κB signaling pathway activation [26]. Collectively, these findings suggest that TRIM38 plays a significant role in the regulation of the innate immune response. Since BspF can mediate the crotonylation of TRIM38 [27], we speculated that BspF might also participate in regulating the host inflammatory pathways.

*Brucella* effector proteins, as substrates of the T4SS, are secreted by the T4SS to act on key cellular signaling pathways, which are relevant to illustrate the process of *Brucella* infection, intracellular survival, and evasion of host-related immune mechanisms. Meanwhile, crotonylation of histones, as a marker of active transcription of some genes, plays an important role in regulating chromatin dynamics, gene expression, and metabolic pathways. In this study, we discovered the role of *Brucella* T4SS effector protein BspF in the infection process, as well as its mechanism of mediating inflammatory pathways, providing valuable research data for further exploring the pathogenesis and treatment of *Brucella*. In addition, this work also provides new references for the functional study of non-histone crotonylation.

## 2. Results

### 2.1. BspF Promotes the Crotonylation of TRIM38

**Figure 1:**
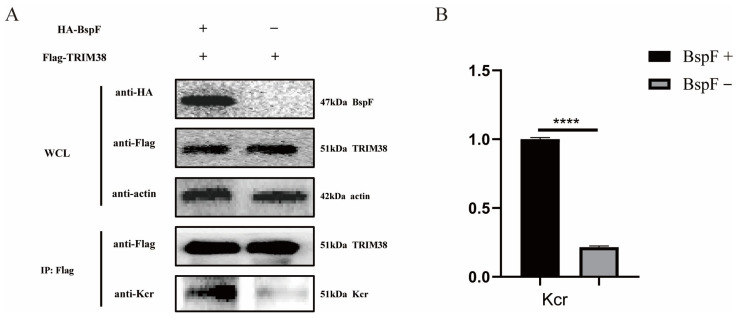
BspF enhanced the crotonylation of TRIM38. (**A**) HA-BspF and FLAG-TRIM38 plasmids were co-transfected into HEK-293T cells and the effect of BspF on TRIM38 crotonylation was detected by Co-immunoprecipitation (Co-IP) with Lysine crotonylation (Kcr), HA-tagged, and FLAG-tagged antibodies. WCL represents the whole cell lysate. (**B**) Densitometric analysis of Western blot bands was performed, and statistical comparisons between the BspF+ and BspF− groups were conducted to evaluate protein expression levels. Data are means ± standard deviation (SD) from three independent experiments. **** indicates *p* < 0.0001.

In our previous study, K142 of TRIM38 was crotonylated by BspF [11]. Therefore, we cloned the gene of TRIM38 and inserted it into the pCMV-Tag2B vector to obtain FLAG-TRIM38 recombinant plasmid, which was co-transfected with pCMV-HA-BspF into HEK-293T cells. The cells were harvested and immunoprecipitated using a FLAG antibody, and the crotonylation of TRIM38 was detected. As shown in Figure 1, the crotonylation of TRIM38 was up-regulated with the expression of BspF.

### 2.2. BspF Inhibits the Activation of Pro-Inflammatory Factors

**Figure 2:**
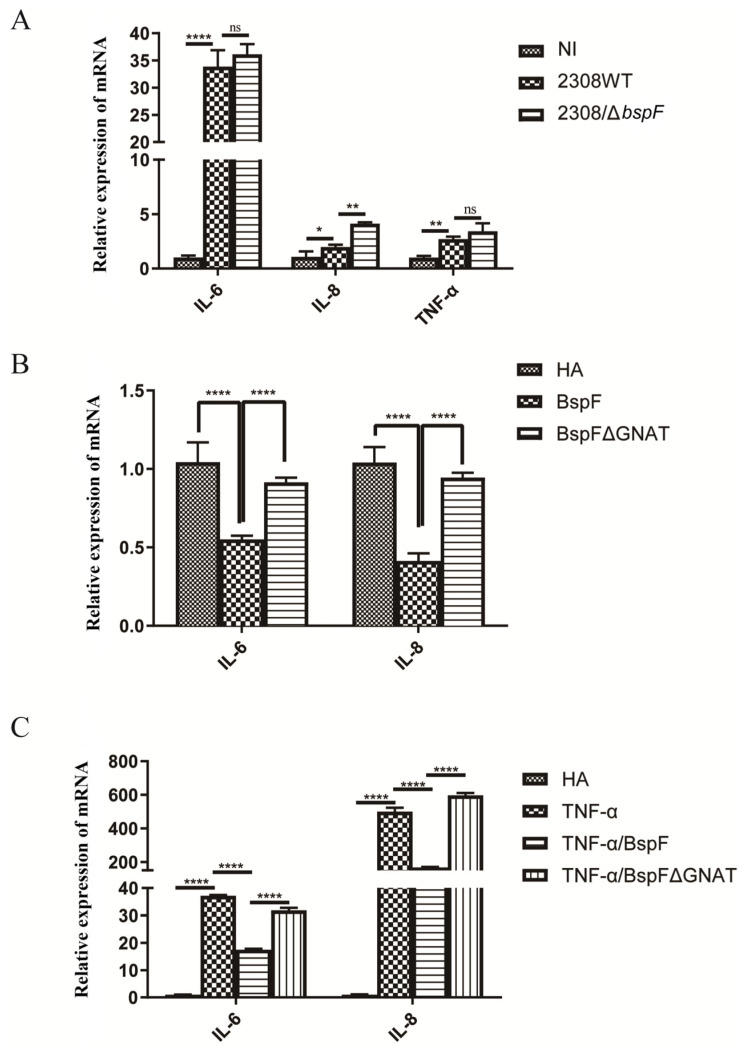
BspF inhibits the transcription of pro-inflammatory factors. (**A**) RAW264.7 cells were infected with 2308WT and 2308bspF strains. After RNA extraction and reverse transcription, *IL-6*, *IL-8*, and TNF-α were detected using RT-PCR. (**B**) HeLa cells were transfected with HA, HA-BspF, and HA-BspFΔGNAT plasmids, and RNA was extracted and reversely transcribed. The mRNA expression of *IL-6* and *IL-8* genes was detected by RT-PCR. (**C**) HeLa cells were transfected with HA, HA-BspF, and HA-BspFΔGNAT plasmids after being treated with TNF-α. The mRNA expression of *IL-6* and *IL-8* genes were detected by RT-PCR. Data are means ± SD from three independent experiments. ns indicates not significant, * indicates *p* < 0.05, ** indicates *p* < 0.01, **** indicates *p* < 0.0001.

TRIM38 is an E3 ubiquitin ligase that negatively regulates TLR3/4-mediated NF-κB activation by targeting TRAF6 for proteasomal degradation to regulate the host’s innate immunity [28]. After verifying the function of BspF in promoting TRIM38 crotonylation, we speculated that BspF may affect the activation of pro-inflammatory factors in host cells. At first, RAW264.7 cells were infected with the wild-type *Brucella* 2308 strain (2308WT) and BspF deletion strain (2308Δ*bspF*). A non-infected (NI) control group was also set up. After the infection, RNA was extracted and reverse transcribed, and the transcription of pro-inflammatory factors *IL-6*, *IL-8*, and Tumor Necrosis Factor-α (TNF-α) was detected with reverse transcript polymerase chain reaction (RT-PCR). The results showed that 2308WT infection promoted the transcription of *IL-6*, *IL-8*, and TNF-α, and compared with 2308WT infection, 2308Δ*bspF* infection increased the transcription of *IL-6*, *IL-8*, and TNF-α, indicating that BspF could inhibit the expression of pro-inflammatory factors in cells during *Brucella* infection (Figure 2A).

In addition, we transfected HA-BspF and HA-BspFΔGNAT expression plasmids into HeLa cells, respectively, when the empty vector (EV) group was set, to observe the effect of BspF on the transcription of pro-inflammatory factors and test whether the transcription of pro-inflammatory factors triggered by BspF depends on the GNAT domain. The results showed that BspF inhibited the transcription of *IL-6* and *IL-8* in HeLa cells. Compared to BspF, the inhibitory effect of BspFΔGNAT on the transcription of pro-inflammatory factors in HeLa cells was weakened (Figure 2B). We preliminarily determined that BspF could inhibit the transcription of pro-inflammatory factors in cells and depended on its GNAT domain. Then, we transfected BspF and BspFΔGNAT expression plasmids after activating the transcription of inflammatory cytokines with TNF-α. As shown in Figure 2C, BspF can still inhibit the transcription of *IL-6* and *IL-8* under the activation by TNF-α.

### 2.3. BspF Inhibits the Activation of Inflammatory Signaling Pathways

**Figure 3:**
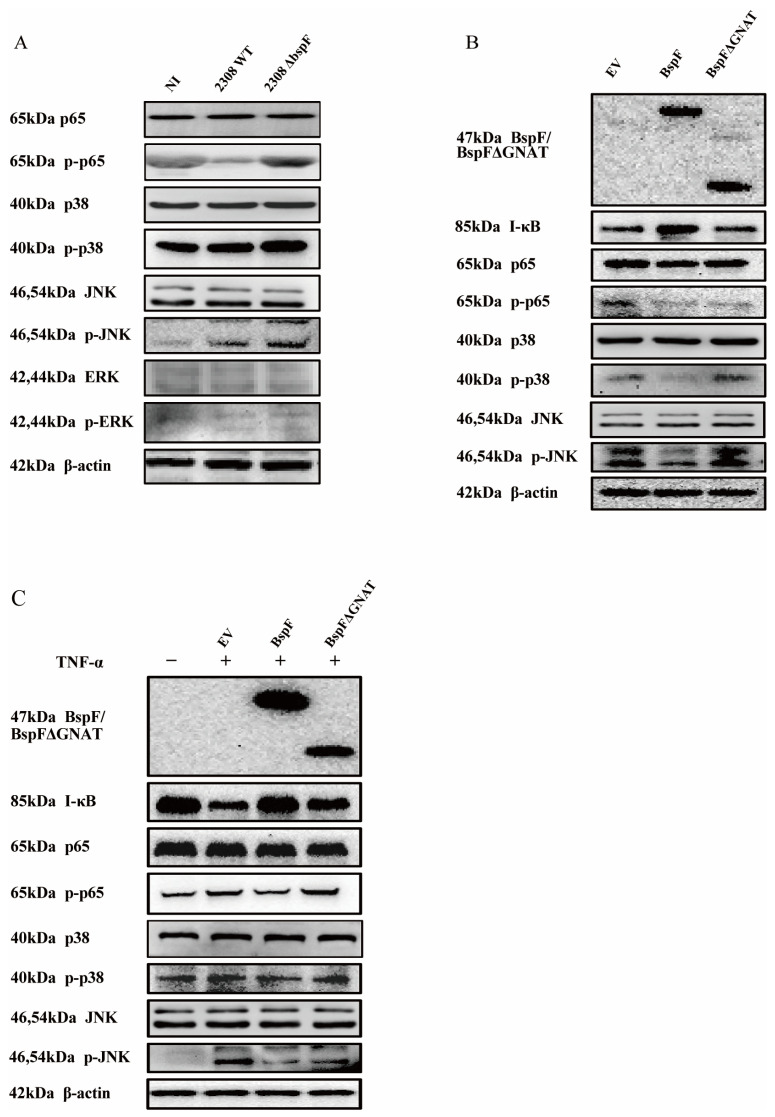
BspF inhibits the activation of NF-κB, p38 MAPK, and JNK MAPK signaling pathways. (**A**) RAW264.7 cells were infected with 2308WT and 2308ΔbspF strains, and the phosphorylation of p65, p38, JNK, and ERK was detected through Western blot. NI indicates uninfected cells. (**B**) HeLa cells were transfected with HA, HA-BspF, and HA-BspFΔGNAT plasmids and the phosphorylation of p65, p38, JNK, and ERK was detected through Western blot analysis. EV indicates empty vector group. (**C**) HeLa cells were transfected with HA, HA-BspF, and HA-BspFΔGNAT plasmids after being treated with TNF-α, and the phosphorylation of p65, p38, JNK, and ERK was detected through Western blot analysis. EV indicates empty vector group. Data are means ± SD from three independent experiments. p-p65, p-p38, p-JNK, p-ERK: phosphorylated forms of the proteins.

Through previous experimental results, we found that BspF relies on the GNAT domain to inhibit the transcription of pro-inflammatory factors, indicating that BspF can inhibit the signal transduction of innate immune pathways during *Brucella* infection. Since the NF-κB pathway and MAPK pathway can be activated by TLR9 and TLR2 signaling pathways after *Brucella* infection [29], we first infected cells with 2308WT and 2308ΔbspF strains, alongside an NI control group, and collected cell samples at 48 h after infection to detect the phosphorylation of p65, p38, JNK, and Extracellular regulated protein kinases (ERK) (p-p65,p-p38, p-pJNK, and p-ERK) in RAW264.7 cells. The results showed that the phosphorylation of p65, p38, and JNK was slightly up-regulated after *Brucella* infection, which meant NF-κB, p38 MAPK, and JNK MAPK pathways were activated. After 2308ΔbspF infection, the phosphorylation levels of p65, p38, and JNK were higher than those of 2308WT infection, indicating that BspF could inhibit the transduction of NF-κB and MAPK signaling pathways during infection (Figure 3A).

In the following experiment, we overexpressed BspF in cells and further scrutinized the effects of the BspF protein on NF-κB, p38 MAPK, and JNK MAPK signaling pathways. Our findings suggest that BspF suppresses the phosphorylation of p65, p38, and JNK. Notably, when the GNAT domain was removed, the suppressive effect was diminished. In summary, our results indicate that the BspF protein can modulate the transduction of NF-κB and p38/JNK signaling pathways via its GNAT domain (Figure 3B,C).

### 2.4. BspF Down-Regulates TRAF6 Protein Expression in a TRIM38-Dependent Manner

**Figure 4:**
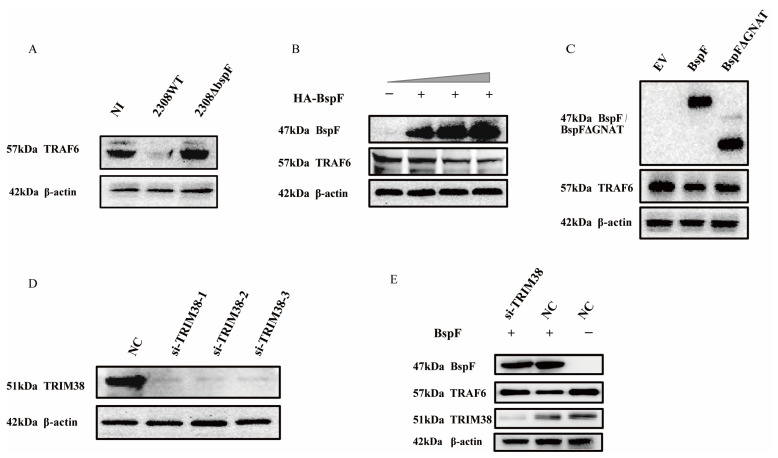
BspF modulates TRAF6 expression via TRIM38. (**A**) RAW264.7 cells were infected with 2308WT and 2308ΔbspF strains, and expression of TRAF6 was detected through Western blot analysis. NI indicates uninfected cells. (**B**) HeLa cells were transfected with different doses of HA-BspF plasmids and TRAF6 expression was detected by Western blot. (**C**) HeLa cells were transfected with HA, HA-BspF, and HA-BspFΔGNAT plasmids and TRAF6 expression was detected by Western blot. EV indicates empty vector group. (**D**) Knockdown effect of the siRNAs at the protein level. HeLa cells were transfected with TRIM38 specific siRNAs and the endogenous TRIM38 protein was assayed through Western blot analysis. NC indicates negative control group. (**E**) HA-BspF plasmid was co-transfected with NC or si-TRIM38 in HeLa cells, and TRAF6 expression was detected through Western blot analysis. Data are means ± SD from three independent experiments.

We have proved the regulation of TRIM38 crotonylation triggered by BspF, and TRIM38 is an E3 ubiquitin ligase that modulated the expression of TRAF6 [26], so we speculated that BspF may regulate NF-κB, p38 MAPK, and JNK MAPK signaling pathways via the regulation of TRAF6 protein expression. Consequently, we examined cellular samples, respectively, infected with 2308WT and 2308ΔbspF, when the NI control group was set, which revealed a considerable reduction in TRAF6 protein expression following 2308WT infection, whereas the protein expression was restored upon 2308ΔbspF infection, supporting the notion that BspF curtails the expression of TRAF6 within cells (Figure 4A). To further probe the effect of BspF on TRAF6 expression in cells, HeLa cells were transfected with varying doses of HA-BspF recombinant plasmid, followed by a TRAF6 expression assessment. Our analysis revealed that TRAF6 expression was inversely correlated with BspF expression (Figure 4B). In subsequent investigation, we overexpressed HA-BspF and HA-BspFΔGNAT recombinant plasmids in cells separately, when the empty vector (EV) group was set, and then detected TRAF6 protein expression through Western blot analysis. The findings demonstrated that the deletion of the GNAT domain resulted in a diminished effect of BspF on TRAF6 protein expression (Figure 4C). Thus, it is evident that BspF influences TRAF6 protein expression via its GNAT domain. In order to substantiate the relation between the effect of BspF on TRAF6 and TRIM38, compared to the negative control (NC) group, TRIM38 was silenced by si-TRIM38 in cells (Figure 4D), followed by the overexpression of BspF. The results indicated that, without silencing the TRIM38 gene, BspF was capable of reducing TRAF6 protein levels within cells. However, following TRIM38 gene silencing, TRAF6 protein expression was restored within cells (Figure 4E). These findings demonstrate that BspF regulates TRAF6 protein expression via TRIM38, in which the GNAT domain plays a pivotal role.

### 2.5. BspF Facilitates TRAF6 Degradation Through Crotonylated TRIM38K142

**Figure 5:**
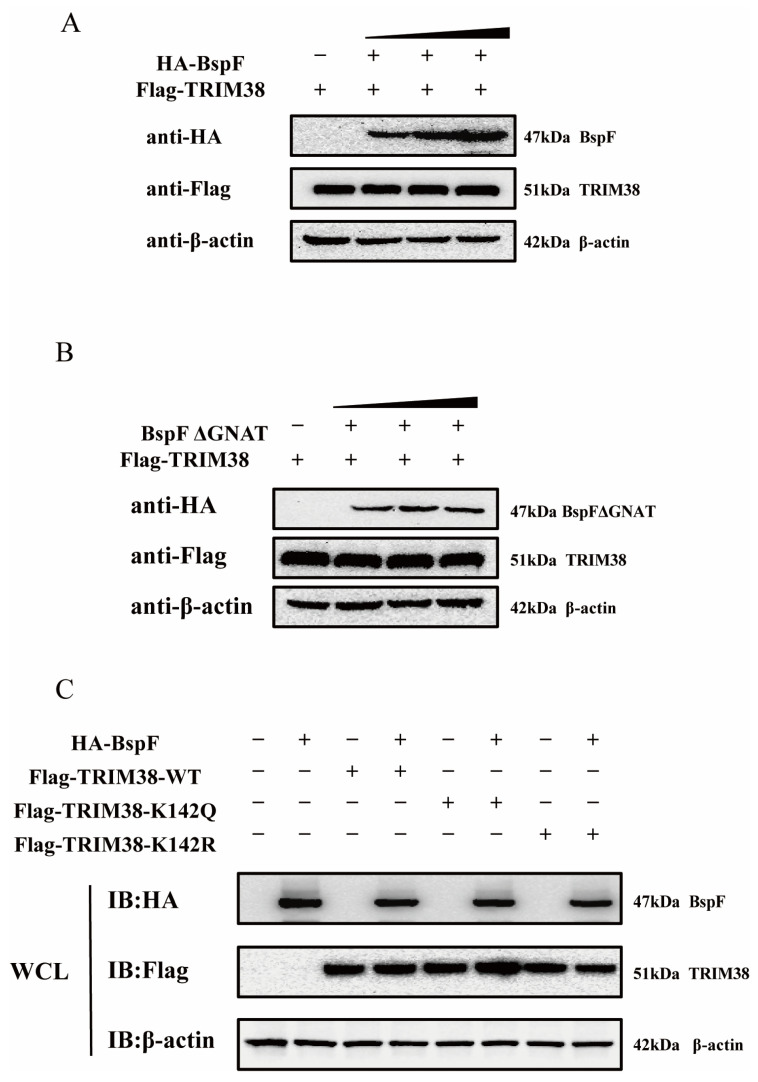
BspF does not affect the TRIM38 expression level through the GNAT domain, and TRIM38K142Cr has no effect on the TRIM38 expression level. (**A**,**B**) Different doses of HA-BspF/BspFΔGNAT and FLAG-TRIM38 plasmids were co-transfected into HEK-293T cells, and the expression of BspF/BspFΔGNAT and TRIM38 was detected through Western blot analysis. (**C**) HA-BspF was co-transfected with the TRIM38 mutant (TRIM38K142R and TRIM38K142Q) into HEK-293T cells and the expression of the TRIM38 mutant (TRIM38K142R and TRIM38K142Q) was detected through Western blot analysis. WCL represents the whole cell lysate. IB represents immunoblotting. Data are means ± SD from three independent experiments.

Owing to the paucity of studies on crotonylation, we surmised that BspF impacts TRIM38 via its GNAT domain in three potential ways. BspF might directly promote TRIM38 protein expression within cells, thereby enabling TRIM38, the E3 ubiquitin ligase, to degrade TRAF6. Another supposition is that BspF might interact with TRIM38, and the BspF-mediated TRIM38K142Cr might affect this interaction, leading to a reduction in TRAF6 expression. Besides these, BspF may influence TRIM38′s function through crotonylation, fostering the degradation of TRAF6 triggered by TRIM38. To confirm our hypothesis, our initial investigation sought to explore whether BspF could directly modulate TRIM38 protein expression. To this end, varying doses of HA-BspF/HA-BspFΔGNAT were co-transfected with FLAG-TRIM38 into cells, and TRIM38 protein expressions were subsequently assayed to visually appraise the impact of BspF/BspFΔGNAT on TRIM38. The results shown in Figure 5A,B suggest that neither BspF nor BspFΔGNAT influence TRIM38 expression within cells.

Additionally, we engineered a TRIM38 mutant (TRIM38K142R and TRIM38K142Q), mutated TRIM38 142 lysine (K) to arginine (R) (TRIM38K142R) transfected cells to mimic the decrotonylated state of the protein, and mutated TRIM38 142 lysine to a glutamine (Q) (TRIM38K142Q) for a protein hypercrotonylation mimic. HA-BspF was co-transfected with FLAG-TRIM38, FLAG-TRIM38K142R, and FLAG-TRIM38K142Q, and we detected the effect of BspF on the expression of the TRIM38 mutant (TRIM38K142R and TRIM38K142Q) with Western blot analysis. The results indicated that BspF did not influence the expression of the TRIM38 mutant (TRIM38K142R and TRIM38K142Q) (Figure 5C). This indicates that BspF does not regulate the expression of TRIM38 by the crotonylation of K142 lysine.

**Figure 6:**
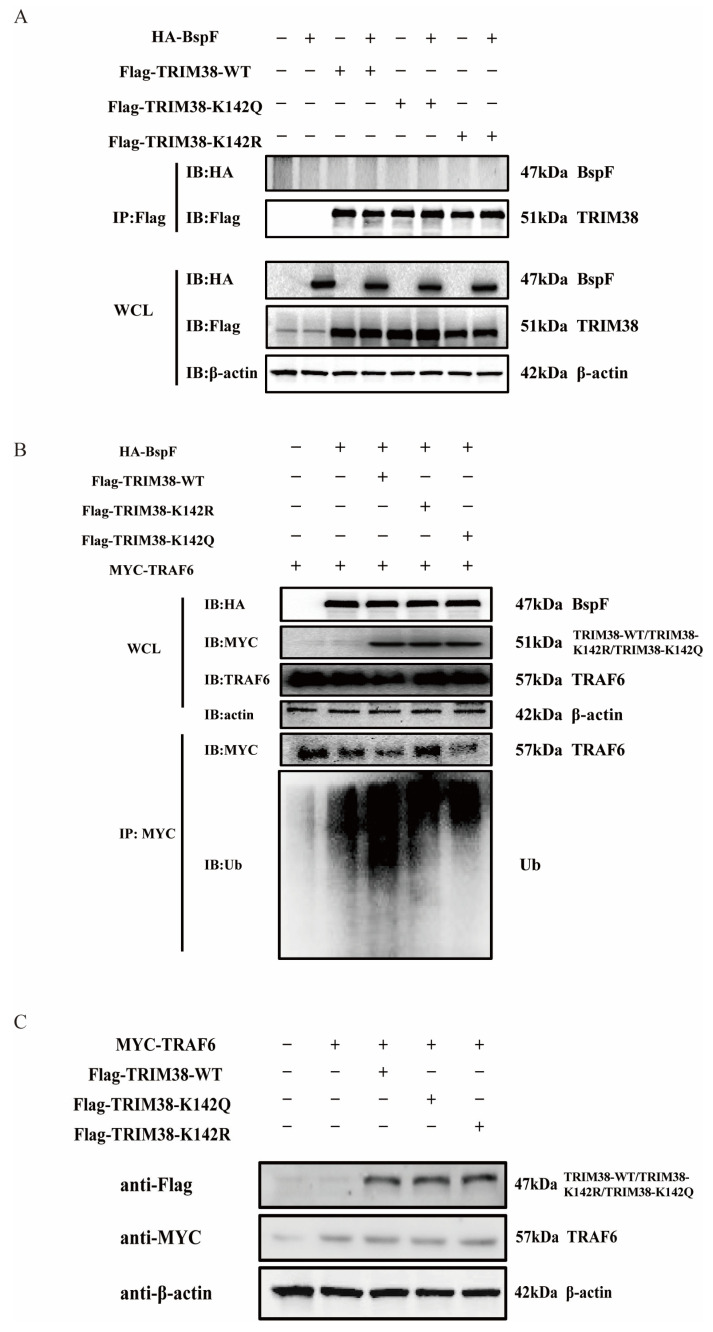
BspF regulates the ubiquitination of TRAF6 through TRIM38K142Cr to affect the degradation of TRAF6. (**A**) HA-BspF was co-transfected with TRIM38 mutant (TRIM38K142R and TRIM38K142Q) plasmids, and the interaction between BspF and the mutant (TRIM38K142R and TRIM38K142Q) was detected with co-immunoprecipitation with FLAG-tagged and HA-tagged antibodies. (**B**) HA-BspF was co-transfected with the TRIM38 mutant (TRIM38K142R and TRIM38K142Q) and MYC-TRAF6 plasmids, performing immunoprecipitation by the MYC antibody, and then the expression and ubiquitination level of TRAF6 was detected through Western blot analysis. (**C**) MYC-TRAF6 was co-transfected with the TRIM38 mutant (TRIM38K142R and TRIM38K142Q) into HEK-293T cells and the expression of the TRIM38 mutant (TRIM38K142R and TRIM38K142Q) was detected through Western blot analysis. Data are means ± SD from three independent experiments.

We then proceeded to test our second hypothesis that BspF interacts with TRIM38, thereby leading to a reduction in TRAF6 protein expression. Toward this objective, HA-BspF was co-transfected, respectively, with FLAG-tagged TRIM38-WT, TRIM38-K142R, and TRIM38-K142Q, and subsequently the protein interaction was examined via immunoprecipitation. Our findings suggested that BspF does not interact with TRIM38, nor does it interact with the TRIM38 mutant (TRIM38K142R and TRIM38K142Q) (Figure 6A). Therefore, we further investigated whether BspF modulated the E3 ubiquitin ligase activity of TRIM38 through TRIM38K142 crotonylation. BspF and TRAF6 were co-transfected with the TRIM38 mutant (TRIM38K142R and TRIM38K142Q), respectively, and the expression and ubiquitination of TRAF6 were assessed. Our results demonstrated that the presence of BspF diminished TRAF6 protein amounts in the cell, and when TRIM38 was also expressed, there was a more significant decrease in TRAF6 expression. Interestingly, the TRAF6 expression in TRIM38K142R transfected cells was higher than that in TRIM38 and TRIM38K142Q overexpressing cells. Correspondingly, the ubiquitination of TRAF6 in TRIM38K142R overexpressed cells was less than that in TRIM38 and TRIM38K142Q transfected cells. These observations provide compelling evidence that BspF modulates cellular TRAF6 protein expression via crotonylation at the K142 site of TRIM38 (Figure 6B), achieving the regulation of inflammatory signaling pathways. In addition, we also co-transfected TRIM38 and its mutants with TRAF6 to observe the degradation of TRAF6 by TRIM38 and its mutants. The results showed no apparent difference in the degradation of TRAF6 by TRIM38 and its mutants in the absence of BspF (Figure 6C).

## 3. Discussion

Building upon prior evidence demonstrating BspF-mediated crotonylation at the K142 site of TRIM38, this study experimentally established that this mechanism enables BspF to coordinately regulate both NF-κB and MAPK signaling pathways, ultimately attenuating host inflammatory responses. Notably, analyses revealed BspF-dependent enhancement of TRIM38 crotonylation levels, which aligns with and extends our previous findings by definitively confirming BspF’s enzymatic capacity as a non-histone crotonyl transferase.

*Brucella* infection enhances activation of pro-inflammatory cytokines including *IL-6*, *IL-8*, IL-10, IL-12, and TNF-α [30,31,32]. Thus, we focused on *IL-6*, *IL-8*, and TNF-α. We found that BspF significantly suppressed their expression, and mechanistic investigations demonstrated that this suppressive effect strictly depended on BspF’s GNAT domain. Notably, while the phenotypic changes observed in transfected HeLa cells were relatively mild compared to *Brucella*-infected RAW cells, this discrepancy likely stems from inherent limitations of transient overexpression systems in replicating complex infection microenvironments. Nevertheless, our data conclusively demonstrate that BspF modulates TRAF6 protein stability through its GNAT domain, thereby regulating host inflammatory responses. To comprehensively validate these findings, we propose the future incorporation of animal infection models to directly assess BspF’s phenotypic impacts on *Brucella* pathogenesis in vivo. Studies have also shown that increased levels of a large number of inflammatory factors, such as *IL-6*, TNF-α, and other inflammatory mediators, can promote platelet activation and predispose patients to thrombosis [33], while BspF in our study can inhibit inflammation. We speculate that BspF can be used as a drug to prevent thrombus formation in the occurrence of some diseases.

In our study, we verified that BspF has de-crotonylase activity in vitro, but it can promote crotonylation in cells. TRAF6 is a key enzyme which functions as a signal transducer in the NF-κB pathway that activates I kappaB kinase (IKK) in response to pro-inflammatory cytokines [33]. TRIM38 can modulate innate immunity as a negative regulator of interferon (IFN)-β production and is a ubiquitin E3 ligase that promotes the ubiquitination of TRAF6 and NLRP3. BspF is an effector protein of *Brucella* that can regulate the intracellular survival of *Brucella*. In this study, BspF was found to affect the degradation of TRAF6 through regulating the crotonylation of K142 site of TRIM38, which can regulate the intracellular inflammatory response. However, the deletion of the GNAT domain increased the intracellular TRAF6 protein content, indicating that the effect of BspF on TRAF6 protein content depends on the presence of the GNAT domain. After silencing the TRIM38 gene in cells, we transfected BspF and observed a significant change in the expression of the TRAF6 protein. The result indicated that the influence exerted by BspF on cellular TRAF6 expression was dependent on TRIM38.

In this study, we propose three hypotheses. Firstly, BspF-mediated crotonylation at TRIM38K142 (TRIM38K142Cr) augments TRIM38 expression, thereby facilitating TRAF6 degradation. Secondly, BspF influences the protein expression of TRAF6 by modulating its interaction with TRIM38K142Cr. Thirdly, BspF amplifies TRIM38 function via TRIM38K142Cr, thereby promoting TRAF6 degradation. However, it is notable that the K142 site does not reside within any recognized domain of TRIM38, but TRIM38K142Cr can enhance the capacity to mediate K48 polyubiquitination of TRAF6. We conjecture that the incorporation of a crotonyl group might alter the three-dimensional conformation of the TRIM38 protein, thereby enhancing its functional potency.

This study provides a new theoretical basis for intracellular survival through the T4SS regulation of innate host immunity and provides an important reference for the study of non-histone crotonylation. We found that *Brucella* BspF promotes the degradation of TRAF6 by mediating TRIM38K142Cr, thereby regulating inflammatory pathways. This means drug development can be conducted for BspF to prevent or mitigate the damage of inflammation during disease. However, the mechanism of BspF regulation of crotonylation was less explored. Additionally, BspF is known to catalyze crotonylation modification in other proteins, such as Rab9A, RAP1B, ATPIF1, USP7, CAND1, and NEDD8, but these mechanisms were not investigated in our study. In response to these limitations, we propose the following solutions: firstly, we plan to enhance our exploration of the mechanism by which BspF regulates crotonylation. Building upon existing studies, we aim to further investigate the role of crotonylation of other proteins catalyzed by BspF in cell function, signal transduction, and disease development. Secondly, we intend to conduct animal experiments and clinical studies to validate the function and mechanism of action of BspF in vivo. This will involve establishing appropriate animal models to mimic the *Brucella* infection process and collecting and analyzing clinical samples to assess BspF expression and activity.

## 4. Materials and Methods

### 4.1. Strains and Cells

In this study, the *Brucella abortus* 2308 (*B. abortus*) wild strain (WT) and 2308 ΔbspF mutant strain were obtained from the Institute of Military Sciences (Beijing, China) and were grown in tryptic soy agar (Solarbio, Beijing, China) and tryptic soy broth (Solarbio, Beijing, China). The template DNA was extracted following the instructions of the Bacterial Genomic DNA Rapid Extraction Kit (Sangon Biotech, Shanghai, China). For gene cloning, Escherichia coli stain DH5α (TransGen Biotech, Beijing, China) was cultured in Luria–Bertani (LB) medium (Solarbio, Beijing, China). HEK-293T, RAW264.7, and HeLa cells were maintained in our laboratory.

### 4.2. Plasmid Cloning

HA-BspF and FLAG-TRIM38 plasmids were preserved by our laboratory [10]. The HA-BspF plasmid was constructed by amplifying the BspF gene from *Brucella* genome using primers BspF-F (AAAGGATCCATGGCTGCAAAACC) and BspF-R (AAACTCGAGTTATTTATGCTCGGTG). The PCR product was gel-purified and cloned into the pCMV-HA vector. The TRIM38 gene was amplified with primers TRIM38-F (AAAGAATTCATGGCCTCAACCACCAG) and TRIM38-R (AAACTCGAGTTAGTCACCTGGGGGAG). Following gel purification, the amplified gene was cloned into the pCMV-Taq2B vector to generate FLAG-TRIM38. The full-length TRAF6 gene was amplified from the HeLa cell genome with PCR using TRAF6-F (AAAGTCGACAATGAGTCTGCTAAAC) and TRAF6-R (AAAGGTACCCTATACCCCTGCATC) primers. After gel purification of the PCR amplified genes, we cloned the TRAF6 gene into the pCMV-MYC vector (MYC-TRAF6).

The primers for the TRIM38 mutants were designed at the website https://tool.vazyme.com:18002/cetool/simple.html (accessed on 6 April 2025). The primers are provided below:

TRIM38K142R-F (CAGCGAGCTGTGACAAAACTGAAGCAACTTGAA),
TRIM38K142R-R (TTTTGTCACAGCTCGCTGGAGCTTTTCCTTGTAGCCC),
TRIM38K142Q-F (AAGCTCCAGCAAGCTGTGACAAAACTGAAGCAAC),
TRIM38K142Q-R (ACAGCTTGCTGGAGCTTTTCCTTGTAGCCCTG).

Subsequently, FLAG-TRIM38 was used as a template, and the plasmid was amplified with the ClonExpress Ultra One Step Cloning Kit V2 point mutation kit (Vazyme, Nanjing, China). The amplified product was digested with DpnI. Afterward, the resulting product was transformed into DH5α competent cells and cultured for 24 h. The colonies were picked for sequencing. Finally, FLAG-TRIM38K142R and FLAG-TRIM38K142Q plasmids were successfully constructed.

### 4.3. Brucella Infection Experiment

RAW264.7 cells were spread on 6-well plates at a density of 1.2 × 10⁶ cells per well and cultured in Dulbecco’s minimal essential medium (DMEM) containing 15% FBS at 37 °C and 5% CO_2_ for 24 h. Then, they were washed three times with phosphate bufferd saline (PBS) and replaced with addition-free DMEM (Servicebio, Wuhan, China). In the P3 laboratory, bacteria cultured at 37 °C for 48 h were collected, divided into 1.5 mL centrifuge tubes, and centrifuged at 12,000 rpm for 1 min. The cells were then washed with PBS three times and resuspended with DMEM. The bacterial suspension was used to infect cells in different wells (MOI = 200). The cells added with bacteria were cultured in a 37 °C incubator for 1 h. Subsequently, the medium containing bacteria was discarded, and the cells were gently washed three times with PBS (Servicebio, Wuhan, China). After that, a cell culture medium containing 2% fetal bovine serum (FBS) (GeminiBio, West Sacramento, CA, USA) and 4% penicillin–streptomycin (or containing 2% FBS and 200 μg/mL gentamicin) was added to the culture dish and incubated at 37 °C for 2 h to kill extracellular bacteria. The medium in the cells was replaced with a cell culture medium containing 2% FBS and 1% penicillin–streptomycin (or containing 2% FBS, 50 μg/mL gentamicin), and the cells were continuously cultured at 37 °C. The medium was removed at 48 h after culturing, and the cells were washed three times with PBS. Trizol (Vazyme, Nanjing, China) was added to lyse cells for RNA collection, while cells were lysed with RIPA (Vazyme, Nanjing, China) buffer for protein sample preparation.

### 4.4. RNA Extraction and Reverse Transcription

Trizol was added to the cells for lysis (1–2 mL Trizol per 1 × 10^7^ cells), fully shaken, and left at room temperature for 5–10 min. Then, chloroform (chloroform/Trizol volume ratio of 1:5) was added. After standing at room temperature for 5 min, it was centrifuged at 12,000× *g* at 4 °C for 15 min. The uppermost solution was transferred to a new RNase-free centrifuge tube, and isopropanol was added. It was left to stand for 10 min at room temperature and then centrifuged at 12,000× *g* and 4 °C for 10 min in a high-speed refrigerated centrifuge. Subsequently, freshly prepared 75% ethanol was added, followed by centrifugation at 7500× *g* and 4 °C for 5 min in a high-speed refrigerated centrifuge, and the supernatant was discarded. The RNA pellet was resuspended in 50 μL RNase-free water immediately upon appearing translucent.

Subsequently, the obtained RNA was reverse transcribed using the HiScript III RT SuperMix for qPCR (+gDNA wiper) kit (Vazyme, Nanjing, China). The synthesized cDNA was either directly used for qPCR amplification or stored at −20 °C for up to six months. For long-term preservation, cDNA was archived at −80°C to avoid repeated freezing and thawing.

### 4.5. Real-Time PCR

The cDNA obtained through reverse transcription was amplified using the 2 × ChamQ SYBR qPCR Master Mix with SYBR Green I dye premix reagent, in a QuantStudio 3 fluorescence quantitative analyzer (Thermo Fisher Scientific, Waltham, MA, USA). The fluorescence detection primers were designed to detect the difference in specific gene expression among different samples. The amplification system of RT-PCR was shown in Table 1. 

Each sample was repeated three times. The fluorescence PCR instrument interface was set as depicted in Table 2. The relative expression levels of *IL-6*, *IL-8*, and TNF-α were calculated by 2^−ΔΔCT^ with β-actin as the reference gene. The primers are provided below:

*IL-6*-F (ACCACTCCCAACAGACCTG), *IL-6*-R (CTGCAAGTGCATCATCGTTG),
*IL-8*-F (GGCCCAATTACTAACAGGT), *IL-8*-R (ATATAGAGGCTTTTCATGCTCA),
TNF-α-F (ATGGCCTCCCTCTCATCAG), TNF-α-R (CTCAGCCACTCCAGCTGCTC).

**Table 2:** RT-PCR reaction conditions.

Stage	Step	Cycle Number	Temperature	Time
Stage 1	Pre—Denaturation	Rep: 1	95 °C	30 s
Stage 2	Cyclic Reaction	Rep: 40	95 °C	10 s
			60 °C	30 s
Stage 3	Dissolution Curve	Rep: 1	95 °C	15 s
			60 °C	60 s
			95 °C	15 s

### 4.6. Cell Culture, Plasmid Transfection

HEK-293T and HeLa cells were cultured in DMEM containing 10% FBS at 37 °C and 5% CO_2_. RAW264.7 cells were cultured in DMEM containing 15% FBS at 37 °C and 5% CO_2_.

To prepare for transfection, plate cells were cultured in 10 mL of 10% growth medium for one day until they reached 70–90% confluency (8 × 10^6^ per well). Lipofectamine 2000 Reagent (Thermo Fisher Scientific, Waltham, MA, USA) was diluted in Opti-MEM Medium (Thermo Fisher Scientific, Waltham, MA, USA), and DNA was also diluted in Opti-MEM Medium and incubated for 10 min. Then, the diluted DNA was added to the diluted Lipofectamine 2000 Reagent in a 1:1 ratio, mixed gently, and incubated for 30 min at room temperature. The resulting DNA–lipid complexes were added to the cells. Six hours after transfection, the growth medium was removed from the cells and replaced with 10 mL of 2% FBS maintenance medium. Cells were incubated at 37 °C in a CO_2_ incubator for 18–36 h post-transfection before assaying for transgene expression.

### 4.7. Western Blot and Co-Immunoprecipitation Experiments

Cells were collected at 30 h after transfection, and then lysed with RIPA (150 mM NaCl, 50 mM Tris-HCl (pH 7.4), 2 mM Disodium ethylenediaminetetraacetate (Na_2_EDTA), 10% glycerol, 1% Nonidet P-40 (NP-40), and 0.1% SDS) and centrifuged in a 1.5 mL centrifuge tube at 12,000 rpm for 10 min at 4 °C, with 2 μL of anti-MYC antibody added to the supernatant for 2 h, followed by 50 μL of Protein A/G Agarose beads at 4 °C for overnight incubation. The Protein A/G Agarose beads were washed three times with the RIPA buffer.

Cell lysates were harvested at 30 h and the protein concentration was determined. Equal amounts of cell lysates were then denatured in 5 × loading buffer and boiled at 100 °C for 10 min. The proteins were then separated by 10% SDS-PAGE and transferred to polyvinylidene fluoride (PVDF) membranes (Merck Millipore, Billerica, MA, USA). The membranes were washed in Tris-buffered saline with Tween 20 (TBST) and blocked in TBST containing 5% skimmed milk for 2 h at room temperature. Next, the membranes were incubated overnight at 4 °C with antibodies. After that, the membranes were incubated with an Horseradish peroxidase (HRP)-conjugated antibody (Beyotime, Shanghai, China) at room temperature for 2 h. Signals were detected with Clarity ECL reagents (Beyotime, China), and the dilution of antibodies was 1:1000.

### 4.8. Antibodies and Other Reagents

We used anti-HA (Medical & Biological Laboratories, Tokyo, Japan), anti-FLAG (MBL, Japan), anti-MYC (Medical & Biological Laboratories, Tokyo, Japan), anti-phosphorylation-p38 (Cell Signaling Technology, Danvers, MA, USA), anti-phosphorylation-p65 (CST, USA), anti-phosphorylation-JNK (CST, USA), anti-phosphorylation-ERK (CST, USA), anti-Ub (CST, USA), anti-p65 (CST, USA), anti-JNK (CST, USA), anti-ERK (CST, USA), anti-p38 (CST, USA), anti-TRAF6 (CST, USA), anti-β-actin (Beyotime, Shanghai, China), anti-Crotonyllysine (Rabbit pAb, Beyotime, Shanghai, China), and HRP-conjugated antibodies (Beyotime, Shanghai, China), as well as Protein A/G Agarose (Beyotime, Shanghai, China).

### 4.9. Statistical Analysis

Each IP or Western blot Western blot assay was repeated independently three times. In this article, Image J (1.8.0) software was used to analyze the results in band densitometry, and the band densitometry results were divided by the band density of the internal reference for homogenization to obtain the final band density. The univariate analysis was used to process this part of the experimental data, and *p* > 0.05 indicates that the difference is not significant, * *p* < 0.05, ** *p* < 0.01, *** *p* < 0.001, **** *p* < 0.0001.

## Figures and Tables

**Table 1:** RT-PCR amplification system.

Reactive Component	Volume (μL)	Concentration
cDNA	1	500 ng/μL
Forward primer	0.5	10 μm
Reverse primer	0.5	10 μm
SYBR Green I Master (2×)	10	
RNase Free H_2_O	8	
Total volume	20	

## Data Availability

The data presented in this study are available upon request from the corresponding author.

## Abbreviations

The following abbreviations are used in this manuscript:

aa	Amino acid
CβG	Cyclic β-1-2-glucan
Co-IP	Co-immunoprecipitation
DMEM	Dulbecco’s Modified Eagle Medium
ERAD	Endoplasmic Reticulum-Associated Degradation
ERK	Extracellular regulated protein kinases,
EV	Empty vector
GNAT	GCN5-Related N-Acetyltransferases
GAP	GTPase activator protein
HIV	Human Immunodeficiency Virus
HRP	Horseradish peroxidase
IFN	Interferon
IKK	I kappaB kinase
IL	Interleukin
JNK	c-Jun N-terminal kinases
Kcr	Lysine crotonylation
kDa	Kilodalton
LPS	Lipopolysaccharide
MAPK	Mitogen-activated protein kinase
MOI	Multiplicity of infection
Na_2_EDTA	Disodium ethylenediaminetetraacetate
NF-κB	Nuclear factor-kappaB
NI	Non-infected
rBCV	replicative *Brucella*-containing vacuole
PBS	Phosphate bufferd saline
RT-PCR	Reverse transcript polymerase chain reaction
SD	Standard Deviation
SDS-PAGE	Sodium dodecylsulphate-polyacrylamide gel electrophoresis
T4SS	Type IV secretion system
TGN	Trans-Golgi network
TLR	Toll-like receptor
TNF-α	Tumor necrosis factor
TNFR	Tumor necrosis factor receptor
TRAF6	TNF receptor-associated factor 6
TRIF	TIR domain-containing adaptor inducing 1FN-β
TRIM38	E3 ubiquitin-protein ligase TRIM38
TRIM38K142Cr	crotonylation at TRIM38K142
Vce	VirB-coregulated effector
WCL	Whole Cell Lysate
